# Multilocus genotyping of *Giardia duodenalis* in captive non-human primates in Sichuan and Guizhou provinces, Southwestern China

**DOI:** 10.1371/journal.pone.0184913

**Published:** 2017-09-14

**Authors:** Zhijun Zhong, Yinan Tian, Wei Li, Xiangming Huang, Lei Deng, Suizhong Cao, Yi Geng, Hualin Fu, Liuhong Shen, Haifeng Liu, Guangneng Peng

**Affiliations:** 1 Key Laboratory of Animal Disease and Human Health of Sichuan Province, College of Veterinary Medicine, Sichuan Agricultural University, Chengdu, Sichuan, P.R. China; 2 Chengdu Research Base of Giant Panda Breeding, Sichuan Key Laboratory of Conservation Biology for Endangered Wildlife, Chengdu, China; Sichuan University, CHINA

## Abstract

*Giardia duodenalis* is a common human and animal pathogen. It has been increasingly reported in wild and captive non-human primates (NHPs) in recent years. However, multilocus genotyping information for *G*. *duodenalis* infecting NHPs in southwestern China is limited. In the present study, the prevalence and multilocus genotypes (MLGs) of *G*. *duodenalis* in captive NHPs in southwestern China were determined. We examined 207 fecal samples from NHPs in Sichuan and Guizhou provinces, and 16 specimens were positive for *G*. *duodenalis*. The overall infection rate was 7.7%, and only assemblage B was identified. *G*. *duodenalis* was detect positive in northern white-cheeked gibbon (14/36, 38.9%), crab-eating macaque (1/60, 1.7%) and rhesus macaques (1/101, 0.9%). Multilocus sequence typing based on beta-giardin (*bg*), triose phosphate isomerase (*tpi*) and glutamate dehydrogenase (*gdh*) revealed nine different assemblage B MLGs (five known genotypes and four novel genotypes). Based on a phylogenetic analysis, one potentially zoonotic genotype of MLG SW7 was identified in a northern white-cheeked gibbon. A high degree of genetic diversity within assemblage B was observed in captive northern white-cheeked gibbons in Southwestern China, including a potentially zoonotic genotype, MLG SW7. To the best of our knowledge, this is the first report using a MLGs approach to identify *G*. *duodenalis* in captive NHPs in Southwestern China.

## Introduction

*Giardia duodenalis* is the etiological agent of giardiasis, a gastrointestinal infection that is typically asymptomatic, but may also be severe in some individuals [1–3]. At present, there are eight distinct assemblages of *G*. *duodenalis* (A-H), assemblages A and B frequently infect humans and animals, assemblages C and D have been described in domestic and wild canines, assemblage E have been widely reported in ruminants but sporadically detected in NHPs and humans, assemblage F in cats, assemblage G in rodents and assemblage H in seals and gulls [4]. Assemblages A and B are considered zoonotic genotypes. In addition to humans, they are widely reported in non-human primates (NHPs) [4–6].

NHPs are valuable wildlife resources. Owing to their high genetic homology to humans, NHPs are important experimental models for clinical research and public health research. *G*. *duodenalis* have a monoxenous life cycle and can spread rapidly in captive NHPs [7]. Genetic polymorphism of *G*. *duodenalis* has been widely investigated in NHPs. Assemblages A, B and E are found in NHPs and assemblage B is dominant [5, 6]. Molecular analyses have revealed that assemblage A is further classified into three major subtypes (AI-AIII), but assemblage B includes many subtypes that have not been systematically categorized [4, 5].

However, little is known about genetic variation in *G*. *duodenalis* infecting NHPs based on multi-locus genotyping. Molecular analyses to date have typically focused on a single genetic locus [4, 8, 9]. Inconsistent genotyping results have sometimes been observed among different individual loci [4, 10]. To better understand the genetic heterogeneity and zoonotic potential of *G*. *duodenalis*, multi-locus genotyping (MLG) employing beta-giardin (*bg*), triose phosphate isomerase (*tpi*) and glutamate dehydrogenase (*gdh*) has been used for genotyping and subtyping *G*. *duodenalis* in humans and animals [11–13]. The aim of the present study was to characterize *G*. *duodenalis* in captive NHPs in Southwestern China. These findings improve our understanding of the genetic diversity and the transmission routes of *G*. *duodenalis* in NHPs.

## Methods

### Ethics statement

This study was reviewed and approved by the Institutional Animal Care and Use Committee of Sichuan Agricultural University under permit number DYY-S20156703. Prior to the collection of fecal specimens from NHPs, permission was obtained from owners.

### Specimen collection

From March to May 2016 and September to November 2016, 207 fecal specimens from NHPs were collected from Sichuan and Guizhou provinces. Fresh fecal specimens were collected immediately after defecation on the ground and separately stored in 50-mL centrifuge tubes. The specimens were kept cool during transport and arrival at the Sichuan Agricultural University. Specifically, 101 samples were obtained from rhesus macaques from the National Experimental Macaque Reproduce Laboratory in Southwest China (n = 31), Chengdu Gaoxin rhesus macaque farm (n = 30), Chengdu zoo (n = 20) and Bifengxia zoo (n = 20). Thirty-six samples were from northern white-cheeked gibbons from zoos in Guiyang (n = 30), Chengdu (n = 2) and Bifengxia (n = 4). Nine samples were from Golden snub-nosed monkeys in the Chengdu zoo. Sixty samples were from crab-eating macaques in the National Experimental Macaque Reproduce Laboratory in Southwest China and the Chengdu Gaoxin rhesus macaque farm (Table 1). Samples were preserved in 2.5% potassium dichromate at 4°C in a refrigerator. All samples were processed within 24 h of collection.

**Table 1:** Prevalence of *Giardia duodenalis* in non-human primates.

Common name (scientific name)	Area	No. tested	No. (%) of positive specimens
Rhesus macaque (*Macaca mulatta*)	National experimental Macaque Reproduce Laboratory in Southwest China	31	0 (0)
	Guiyang zoo	20	1 (5)
	Bifengxia zoo	20	0 (0)
	Chengdu Gaoxin rhesus macaque farm	30	0 (0)
Northern white-cheeked gibbon (*Nomascus leucogenys*)	Guiyang zoo	30	14 (46.7)
	Chengdu zoo	2	0 (0)
	Bifengxia zoo	4	0 (0)
Golden snub-nosed mokey (*Rhinopithecus roxellanae*)	Chengdu zoo	9	0 (0)
Crab-eating macaque (*Macaca fascicularis*)	Ya`an rhesus macaque base	30	0 (0)
	Chengdu Gaoxin rhesus macaque farm	30	1 (3.3)

### DNA extraction and PCR amplification

Before extracting DNA, the fecal samples were washed with distilled water until potassium dichromate was removed. Genomic DNA was extracted using the PowerSoil^®^ DNA Isolation Kit (MoBio, Carlsbad CA, USA) following the manufacturer`s instructions. DNA samples were stored in 100 μL of the kit's Solution Buffer at 20°C until use.

Each specimen was examined for *G*. *duodenalis* by nested PCR amplification of the beta-giardin (*bg*) gene [14], The *bg*-positive specimens were further characterized by PCR amplification of the *tpi* and *gdh* genes [11]. Secondary PCR products were visualized by staining with Golden View following 1% agarose gel electrophoresis.

### Sequencing and phylogenetic analysis

The amplified products of the expected size were sequenced by Invitrogen (Shanghai, China). To determine the *G*. *duodenalis* assemblage, the sequences were aligned with sequences downloaded from the GenBank database based on a BLAST analysis (http://blast.ncbi.nlm.nih.gov) using ClustalX. For the phylogenetic analysis, sequences obtained in this study were used to construct a neighboring-joining tree using Mega 5 (http://www.megasoftware.net/). A total of 1000 replicates were used for the bootstrap analysis.

### Statistical analysis

Differences in infection rates among NHPs and among animals in different areas were assessed using the chi-square test implemented in SPSS version 17.0 (SPSS Inc., Chicago, IL, USA). P < 0.05 was considered significant.

## Results and discussion

In the *bg*-based PCR analysis of 207 specimens from 4 NHP species, 16 (7.7%) samples from 3 species were positive for *G*. *duodenalis*. All the positive specimens were successfully amplified and sequenced for the *bg*, *tpi* and *gdh* genes. Sequences were deposited in the GenBank database under the accession numbers KY696790-KY696821.

The infection rates ranged from 0% to 38.9% in the 4 species (Table 1). Specifically, 1 of 101 (0.9%) rhesus macaques and 1 of 60 (1.7%) crab-eating macaques were positive for *G*. *duodenalis*. Northern white-cheeked gibbons showed the highest infection rate (14/36, 38.9%). All golden snub-nosed monkeys (n = 9) were negative for *G*. *duodenalis*. The difference in infection rates among 4 species was significant (P<0.05). In China, six studies have examined *G*. *duodenalis* infection in NHPs in parks, zoos, farms and laboratories to date, and the overall infection rate was between 1.3% and 18.6% in these studies [7, 15–19]. The overall infection rate in our study (7.7%) was close to the total infection rate in Qianling Park in Guiyang (8.5%) [17], and was much lower than the total prevalence in zoos in China (18.6%) [15]. It was obviously higher than those reported in Guangxi (2.4%) [19], Qinling Mountain (2.0%) [16] and two other additional comprehensive parasite infection studies in China (2.2% and 1.3%) [7, 18]. Our results and those of previous studies indicate that *G*. *duodenalis* infection is common in wild and captive NHPs and has a wide geographic distribution in China.

In other countries, *G*. *duodenalis* infection in NHPs showed a similar trend to that observed in China. The overall infection rate of *G*. *duodenalis* in NHPs is between 2.2% and 47.0% [7, 20], indicating a wide range of infection rates. The prevalence in our present study was close to previous estimates in Italy (6.0%) [20] and Thailand (7.0%) [6], and it was lower than the infection rates reported in Uganda (11.1%) [21] and Croatia (50%) [22]. This result may be explained by differences among regions in climate, environmental management, NHPs species and animal exchange programs [5, 8, 20].

In this study, the infection rate for captive NHPs in Sichuan province was 0.6% (1/106), which is almost identical to that in a comprehensive parasite study performed in 2009–2015 in Sichuan (0.5%, 3/581) [18]. The infection rate in captive NHPs in Guizhou province was 30% (15/50), much higher than that of free-range NHPs in Guiyang (8.5%, 35/411) [17]. Additionally, 38.9% (14/36) of northern white-cheeked gibbons were positive for *G*. *duodenalis*, which was also higher than the infection rate in a previous study (14.3%, 2/14) [15]. These results suggest that captive northern white-cheeked gibbons are more prone to infection by *G*. *duodenalis* than wild animals. This might be explained by the single-host life cycle and the resilient infectious cysts of *G*. *duodenalis* [23]. Captive northern white-cheeked gibbons are closer to each other than free-range NHPs, and confined spaces result in the transmission of infectious cysts between NHPs. The high transmission between captive NHPs is consistent with those of previous studies in China [7, 15].

To date, assemblages A, B and E have been detected in NHP species in China. Assemblage A and B have both been found in captive and free-range NHPs, but assemblage E has only been found in captive NHPs [16]. In this study, only assemblage B was detected in 3 captive NHP species, consistent with a recent study in zoos in China [7]. In these previous studies, all specimens were obtained from captive NHPs inhabiting in zoos, farms or bases. The resilient infectious cysts of *G*. *duodenalis* may explain the low infection diversity of assemblages [4], and suggests that assemblage B is predominant in Sichuan province. Assemblage B was identified in rhesus macaques, northern white-cheeked gibbons and crab-eating macaques. According to a previous study in 2009–2015 [15], assemblage B was only identified in rhesus macaques and northern white-cheeked gibbons, but assemblages A and B were both identified in crab-eating macaques. This result may suggest that northern white-cheeked gibbons are more susceptible to assemblage B than to other assemblages, and assemblage A might be host-specific including few NHPs species.

No genetic variation was observed among the 16 *gdh* sequences. All of the 16 sequences were 100% similar to sequences of human isolates from Brazil (one strain was identical to EF507672 and 15 strains were identical to EF507682) [24]. The *bg* and *tpi* loci showed high levels of sequence polymorphism. 5 and 6 subtypes were identified in the 16 strains, including two new subtypes. At the *bg* locus, four known subtypes Bb-4 (KJ888977) [15], BIII (KF922976) [25], BIII-1 (EU637581) [26], and B1 (KM211793) [27] were found in 1, 7, 2 and 1 specimens, respectively. The novel subtype, named BIII-2, was found in 5 specimens in our study. At the *tpi* locus, five known subtypes B7 (JQ863259) [28], WB8 (KF679738) [7], EB5 (KT948110) [13], BIV (AB618783) [29] and WB6 (KJ888987) [15] were found in 1, 7, 1, 3 and 1 specimens, respectively. The novel subtypes, named B9, was found in 3 specimens (Table 2).

**Table 2:** Characterization of 16 specimens based on multi-locus sequences of *bg*, *tpi* and *gdh* genes.

Isolate	Host	Geographic source	subtype/host or source/GenBank accession number			MLG
			*bg*	*tpi*	*gdh*	
YA053	Rhesus macaque	National experimental Macaque Reproduce Laboratory in Southwest China	Bb-4/Lemur catta/ KJ888977	B7/wastewater/JQ863259	BIV/Human/EF507672	SW1
GY004	Northern white-cheeked gibbon	Guiyang zoo	BIII/Human/KF922976	WB8/rhesus macaque/KF679738	BIV/Human/EF507682	SW2
GY006	Northern white-cheeked gibbon	Guiyang zoo	BIII-2/ northern white-cheeked gibbon /KY696824^#^	WB8/rhesus macaque/KF679738	BIV/Human/EF507682	SW3*
GY007	Northern white-cheeked gibbon	Guiyang zoo	BIII-2/ northern white-cheeked gibbon /KY696825^#^	WB8/rhesus macaque/KF679738	BIV/Human/EF507682	SW3*
GY013	Northern white-cheeked gibbon	Guiyang zoo	BIII/Human/KF922976	B9/ northern white-cheeked gibbon /KY696810^#^	BIV/Human/EF507682	SW4*
GY014	Northern white-cheeked gibbon	Guiyang zoo	BIII/Human/KF922976	WB8/rhesus macaque/KF679738	BIV/Human/EF507682	SW2
GY015	Northern white-cheeked gibbon	Guiyang zoo	BIII-2/ northern white-cheeked gibbon /KY696828^#^	B9/ northern white-cheeked gibbon /KY696812^#^	BIV/Human/EF507682	SW4*
GY019	Northern white-cheeked gibbon	Guiyang zoo	BIII/Human/KF922976	EB5/Human/KT948110	BIV/Human/EF507682	SW5
GY020	Northern white-cheeked gibbon	Guiyang zoo	BIII/Human/KF922976	WB8/rhesus macaque/KF679738	BIV/Human/EF507682	SW2
GY021	Northern white-cheeked gibbon	Guiyang zoo	BIII/Human/KF922976	WB8/rhesus macaque/KF679738	BIV/Human/EF507682	SW2
GY025	Northern white-cheeked gibbon	Guiyang zoo	BIII-2/ northern white-cheeked gibbon /KY696832^#^	BIV/Human/AB618783	BIV/Human/EF507682	SW6*
GY028	Northern white-cheeked gibbon	Guiyang zoo	BIII-1/Barbary macaque/EU637581	BIV/Human/AB618783	BIV/Human/EF507682	SW7
GY031	Northern white-cheeked gibbon	Guiyang zoo	BIII-1/Barbary macaque/EU637581	BIV/Human/AB618783	BIV/Human/EF507682	SW7
GY033	Northern white-cheeked gibbon	Guiyang zoo	BIII/Human/KF922976	WB8/rhesus macaque/KF679738	BIV/Human/EF507682	SW2
GY034	Northern white-cheeked gibbon	Guiyang zoo	BIII-2/ northern white-cheeked gibbon /KY696836^#^	B9/ northern white-cheeked gibbon /KY696820^#^	BIV/Human/EF507682	SW8*
CD010	Crab-eating macaque	Chengdu Gaoxin rhesus macaque farm	B1/rhesus macaque/KM211793	WB6/Mandrill/KJ888987	BIV/Human/EF507682	SW9

Note:

“^#^”was the novel subtypes;

“*” was the novel MLGs

**Fig 1:**
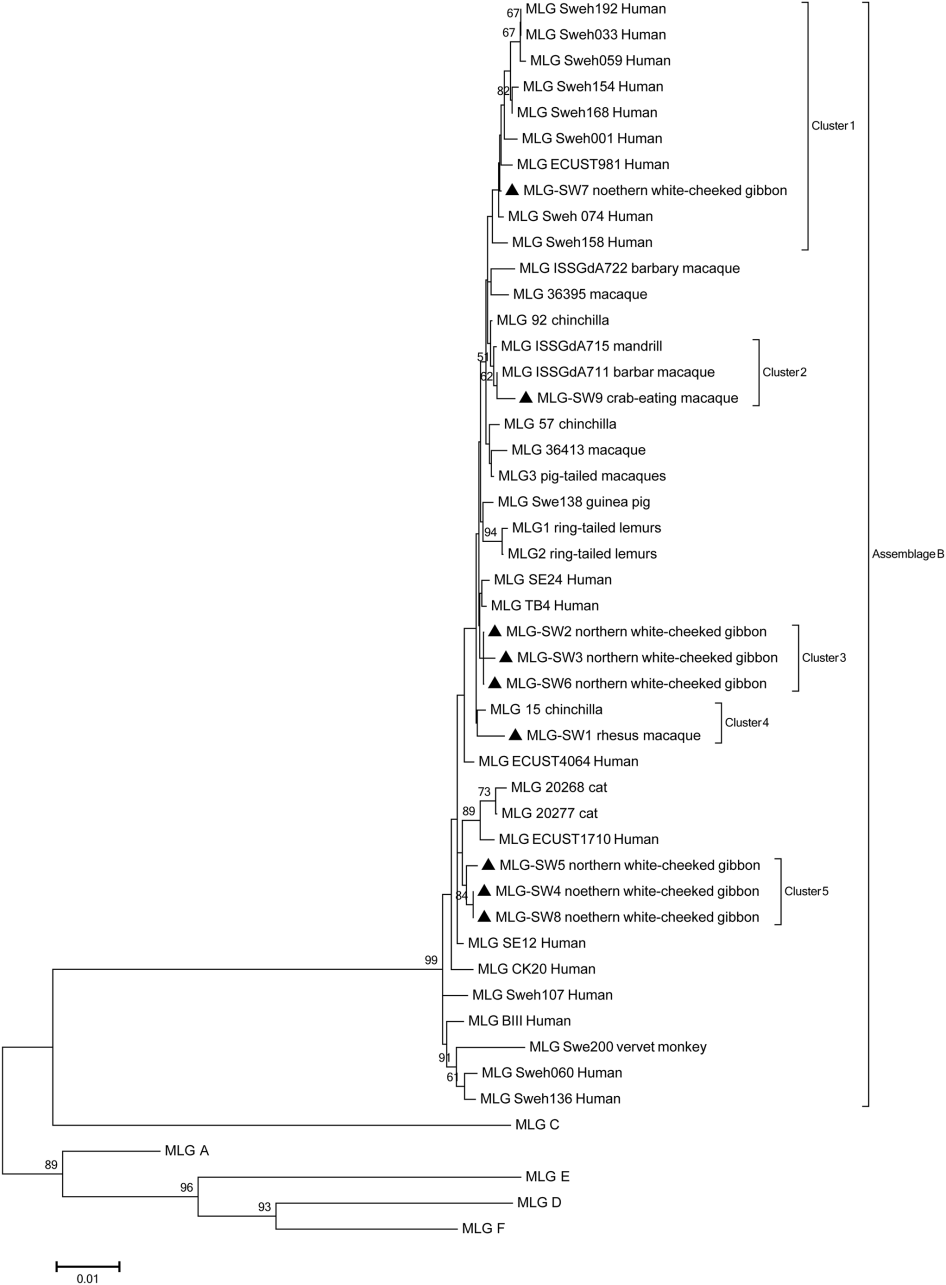
Phylogenetic relationship of *Giardia duodenalis* assemblage B MLGs inferred by the neighbor-joining analysis of concatenated *bg*, *tpi* and *gdh* sequences. Bootstrap values greater than 50% from 1000 replicates are shown. Concatenated sequences from this study are marked by filed roundness.

A total of 16 NHPs specimens (one from a rhesus macaque, one from a crab-eating macaque, and fourteen from northern white-cheeked gibbons) were classified as assemblage B and nine MLGs were identified among the 16 positive specimens. The subtype identities and geographical and host distributions of the nine MLGs are listed in Table 2. A phylogenetic analysis of the concatenated sequences of assemblage B revealed that nine MLGs in this study formed five clusters. MLG SW7, SW9 and SW1 were distributed in three separate clusters. SW 2, 3 and 6 isolated from northern white-cheeked gibbons formed cluster 3. Cluster 5 included SW4, 5 and 8, all of which were isolated from northern white-cheeked gibbons (Fig 1).

Phylogenetic analyses showed that MLG SW7 belonged to a zoonotic group. Given the zoonotic potential of this subtype, epidemiological and source tracking investigations as well as strict surveillance in captive NHPs in southwestern China are needed. MLG SW9 was closely related to the sequences obtained from NHPs in other studies [11]. MLG SW1 was similar to the sequences isolated from chinchillas, suggesting the potential for transmission of *G*. *duodenalis* between animals [16]. Other MLGs formed two separate clusters. In this study, most MLGs (7 MLGs) were found in northern white-cheeked gibbons suggesting greater genetic heterogeneity in *G*. *duodenalis* from this species [15].

## Conclusion

The results of the present study confirm previous findings that assemblage B is dominant in northern white-cheeked gibbons. We first used a MLGs approach to identify *G*. *duodenalis* in captive NHPs in Southwestern China. One genotype of the potentially zoonotic assemblage B of MLG SW7 strain was identified in a northern white-cheeked gibbon. This suggests that the zoonotic transmission of *Giardia* might occur between the northern white-cheeked gibbon and humans. Additionally, high degree genetic diversity of assemblage B MLGs (7 MLGs) was detected in captive northern white-cheeked gibbons in Southwestern China. Additional MLGs studies of captive NHPs are needed, to better characterize genetic diversity and the routes of transmission of *G*. *duodenalis* between NHPs and humans or other animals.
