# Correction: Multilocus genotyping of *Giardia duodenalis* in captive non-human primates in Sichuan and Guizhou provinces, Southwestern China

**DOI:** 10.1371/journal.pone.0194584

**Published:** 2018-03-14

**Authors:** Zhijun Zhong, Yinan Tian, Wei Li, Xiangming Huang, Lei Deng, Suizhong Cao, Yi Geng, Hualin Fu, Liuhong Shen, Haifeng Liu, Guangneng Peng

There is an error in the last sentence of the first paragraph in the Results section. The correct sentence is: Sequences were deposited in the GenBank database under the accession numbers KY696790-KY696837.
